# Biochemical characterisation of a collagenase from *Bacillus cereus* strain Q1

**DOI:** 10.1038/s41598-021-83744-6

**Published:** 2021-02-18

**Authors:** Isabel J. Hoppe, Hans Brandstetter, Esther Schönauer

**Affiliations:** Department of Biosciences, University of Salzburg, 5020 Salzburg, Austria

**Keywords:** Biochemistry, Biotechnology, Microbiology, Molecular biology, Structural biology

## Abstract

Collagen is the most abundant protein in higher animals and as such it is a valuable source of amino acids and carbon for saprophytic bacteria. Due to its unique amino acid composition and triple-helical tertiary structure it can however only be cleaved by specialized proteases like the collagenases secreted by some bacteria. Among the best described bacterial collagenases are ColG and ColH from *Clostridium histolyticum*. Many *Bacillus* species contain homologues of clostridial collagenases, which play a role in some infections caused by *B. cereus*. Detailed biochemical and enzymatic characterizations of bacillial collagenases are however lacking at this time. In an effort to close this gap in knowledge we expressed ColQ1 from *B. cereus* strain Q1 recombinantly, investigated its metal dependency and performed peptide, gelatin and collagen degradation assays. Our results show that ColQ1 is a true collagenase, cleaving natively folded collagen six times more efficiently than ColG while at the same time being a similarly effective peptidase as ColH. In both ColQ1 and ColG the rate-limiting step in collagenolysis is the unwinding of the triple-helix. The data suggest an orchestrated multi-domain mechanism for efficient helicase activity.

## Introduction

Collagen is the most abundant protein in mammalian bodies and makes up approximately 30% of the total protein content^1^. It is found in tendons, bone, cartilage, ligaments and skin, as an integral part of the extracellular matrix. There are numerous types of collagen, which all contain a characteristic right-handed triple helix composed of three left-handed α-chains and type I—the most abundant form of collagen—is almost exclusively made up of this triple helix. The helical structure is supported by a glycine at every third amino acid position and a high content of proline and hydroxyproline^1–3^.

This unusual structure and the high content of imino acids give collagen a remarkable tensile strength and a high resistance to proteolysis^4^. Even endogenous collagenases like MMP-1 can only cleave native collagen at one single site^5^. In contrast to that, bacterial collagenases can cleave collagen at multiple sites and ultimately degrade it into small peptides^6,7^. The best-described bacterial collagenases are ColG and H from *Clostridium histolyticum*. They are large (≈115 kDa) multi-domain proteins consisting of an N-terminal collagenase unit (CU) which in turn is subdivided into the activator domain (AD) and the peptidase domain (PD)^8,9^. The collagenase unit is followed by a varying composition of accessory domains that are involved in substrate recognition and collagen swelling^10,11^.

The peptidase domain contains the catalytic zinc, coordinated by the two histidine residues in the HEXXH motif and a downstream glutamate characteristic for gluzincins^12–14^. It can be further divided into a catalytic and a helper subdomain. The catalytic subdomain is again comprised of an upper and a lower half-domain, with the catalytic zinc ion located at the interface of the two^15,16^.

The primary function of bacterial collagenases is the degradation of collagen as an amino acid and nitrogen source, and as such they enable the recycling of collagen within the global nitrogen cycle^17,18^. They are however also relevant as virulence factors in some diseases such as gas gangrene (*C. perfringens*)^6^ or botulism (*C. botulinum*)^6^, and therefore present interesting targets for novel antibiotics^19^. Furthermore, collagenases isolated from *C. histolyticum* are in medical and biotechnological use as wound debridement agents^20^, treatment for Dupuytren’s contracture^21,22^, islet cell isolation^23–25^ and in the food and leather industry^26,27^.

In contrast to clostridial collagenases, there is only little known about collagenolytic enzymes from bacilli. It has been shown that, similarly to clostridia, bacilli can secrete collagenases into the culture medium^28–30^ and these can play a role in pathogenesis. *Bacillus thuringiensis*, a widely used bacterial insecticide, utilizes ColB for host invasion^31^ and it has been observed that collagenases play a role in *B. cereus* mediated endophthalmitis^32^ and periodontal disease^33^. However, to our knowledge there is no detailed enzymatic or structural data available on collagenases from bacilli. We found two putative collagenases (by homology to ColG from *C. histolyticum*) within the genome of the *B. cereus* strain Q1, reportedly an extremophilic organism^34^. In an effort to close the gap in knowledge about bacillial collagenases, we chose to recombinantly express and characterize one of them (UniProt accession B9J3S4), termed ColQ1 from here on.

## Results

### Bioinformatic analysis

**Figure 1:**
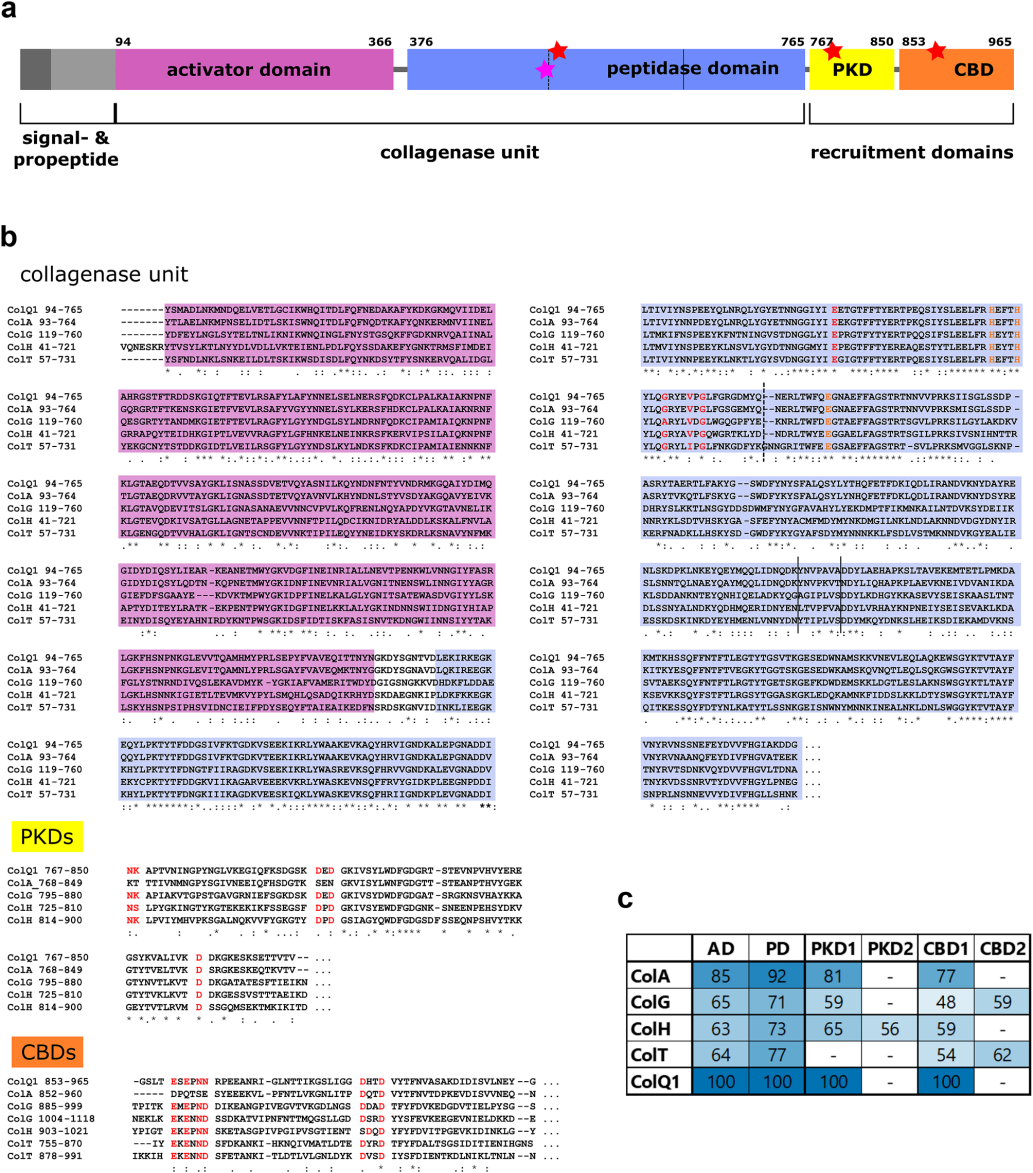
Bioinformatic analysis of ColQ1 (**a**) Schematic domain architecture of ColQ1 based on sequence homology to microbial collagenases and conserved domain predictions^54,55^. Pink star indicates the zinc-binding site and red stars indicate the conserved calcium-binding sites. (**b**) Multiple alignment of mature sequences of ColQ1 (B9J3S4) from *B. cereus* strain Q1, ColA (Q81BJ6) from *B. cereus* strain ATCC 14579, ColG (Q9X721) and ColH (Q46085) from *C. histolyticum* and ColT (Q899Y1) from *C. tetani.* Magenta, light blue, orange and red are the activator domain, peptidase domain, zinc-binding residues and calcium-binding residues^73,74^, respectively. Division of the peptidase domain into the catalytic and the helper subdomain is indicated by solid lines. Division of the catalytic domain into the upper and lower half-domain is indicated by a dotted line in (a) and (b). The alignment was aided by Clustal Omega^75^. (**c**) Similarity [%] of the activator (AD), peptidase (PD), polycystic kidney disease like (PKD) and collagen-binding domains (CBD) of ColA, G, H and T to the corresponding domains in ColQ1. Darker blue indicates higher similarity. Calculated by BLASTp^56^.

Protein sequence alignment using ClustalOmega^35^ of ColQ1 with the clostridial collagenases ColG, H and T and ColA from *B. cereus* strain ATCC 14579 showed high sequence identities of approximately 45% to the clostridial collagenases and over 70% to the bacillus collagenase ColA. Consistent with previous studies^16^, the analysis with SignalP and conserved domains databases predicted the presence of (1) an N-terminal 30 amino acid signal sequence for extracellular secretion, (2) a propeptide (31–93) with unclear function, (3) an M9-like domain (94–366), which functions as an activator domain for collagenolysis (but as nuclear transport receptor in eukaryotes)^16,36^, (4) a peptidase M9 family domain (376–765; referred to as peptidase domain), (5) one polycystic kidney disease (PKD)-like domain (767–850) and (6) one collagen-binding domain (CBD, 853–965), Fig. 1a^37–39^. Domain-wise sequence alignments (Fig. 1b) showed that the similarities are clustered to the peptidase domain (PD) and that the similarity to the bacillial collagenase ColA is much higher than to the three clostridial collagenases (Fig. 1c). The catalytic HEXXH motif and the downstream glutamic acid as third zinc-binding residue that are characteristic for zinc-metalloproteases of the peptidase family M9 are conserved, as are the calcium-binding residues in the peptidase domain and accessory domains (Fig. 1b, indicated in orange and red respectively). The peptidase domain of clostridial collagenases can be further divided into a helper and a catalytic subdomain, with the latter being made up of an upper and a lower half-domain^15^. The boundaries between these subdomains are indicated by solid lines (catalytic and helper subdomains) and dashed lines (upper and lower catalytic half-domain) in Fig. 1a,b. For ColQ1 residues Y94-K965 therefore represent the mature, full-length enzyme, residues Y94-G765 the collagenase unit, Y94-N366 the activator domain (AD) and L376-G765 the peptidase domain (PD).

### Recombinant expression and purification of ColQ1

Based on the production strategy for clostridial collagenases^40^, the ColQ1 variants were recombinantly expressed in *E. coli* NiCo21(DE3) cells and purified by immobilized metal affinity chromatography (IMAC) using an N-terminal His_6_-tag, followed by tag removal via tobacco etch virus (TEV) protease, rechromatography and size exclusion chromatography as a final polishing step.

**Figure 2:**
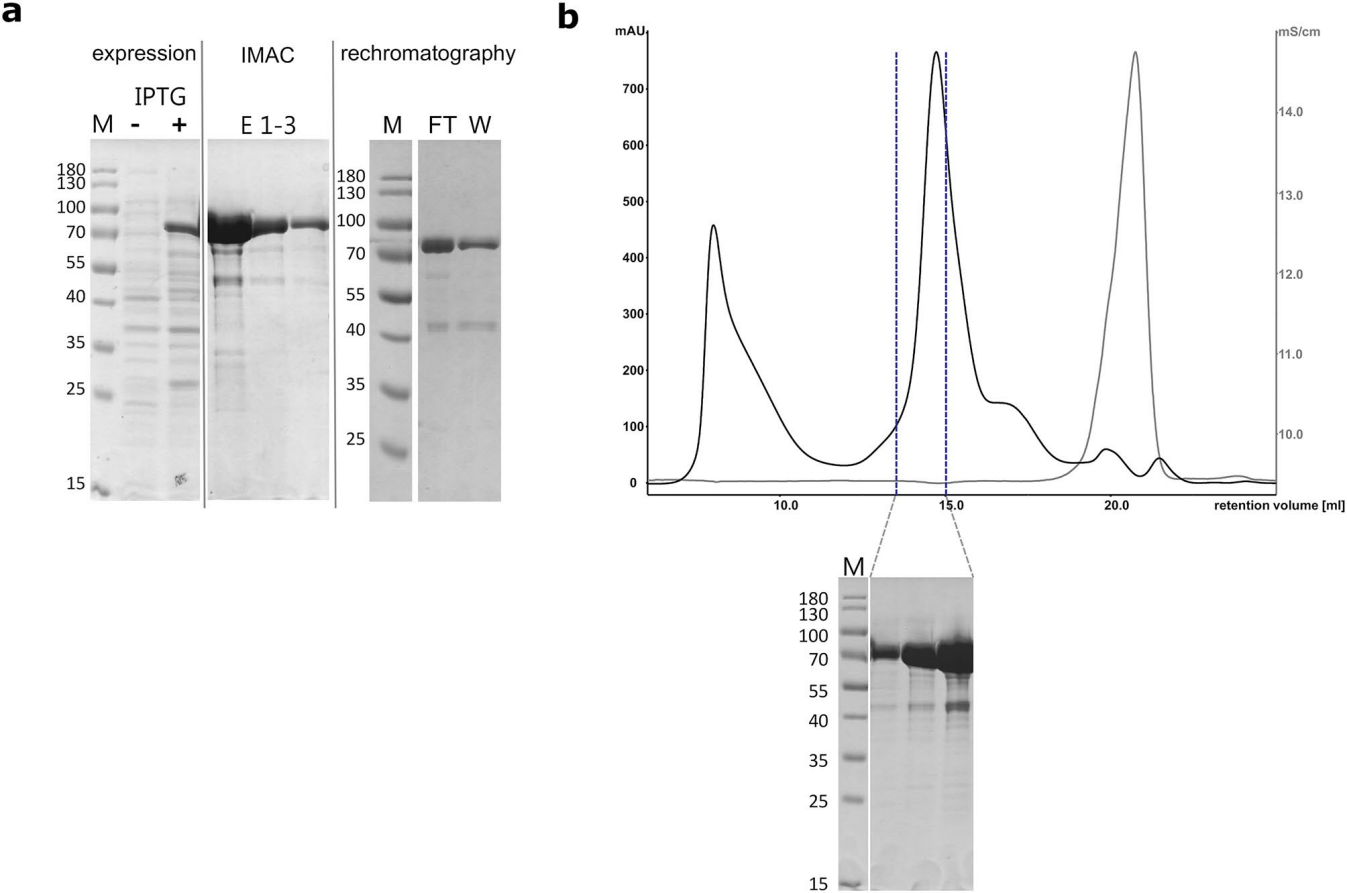
Representative purification of ColQ1 CU (**a**) Expression, IMAC and removal of His_6_-tag. M: molecular weight standards, IPTG ∓ : total cell lysate before induction and at time of harvest, E1-3: elution with 250 mM imidazole, rechromatography FT (flow-through) and W (wash): fractions containing target protein after His_6_-tag cleavage by TEV-protease. (**b**) Size exclusion chromatography and SDS-PAGE of pure protein fractions. The blue dotted lines indicate the fractions that were analysed in the depicted SDS-PAGE gel and subsequently pooled for further use. M: molecular weight standards.

All ColQ1 variants (Y94-K965: full-length mature FL, Y94-G765: CU, E502A dead mutant of both, Y94-N366: AD, L376-G765: PD) were successfully overexpressed in *E. coli* NiCo21(DE3) cells in soluble form and purified by IMAC (Fig. 2a). After tag removal we continued with the fractions that contained cleaved protein (flow-through and wash) to size exclusion chromatography. A considerable amount of aggregated target protein and other contaminants was removed in this step (Fig. 2b). The final product was eluted at the expected retention time for its molecular weight and was estimated by SDS-PAGE to be at least 90% pure at a yield of 5–10 mg target protein per litre of cell culture.

### Thermal stability of ColQ1 compared to the clostridial collagenases ColG and ColH

**Figure 3:**
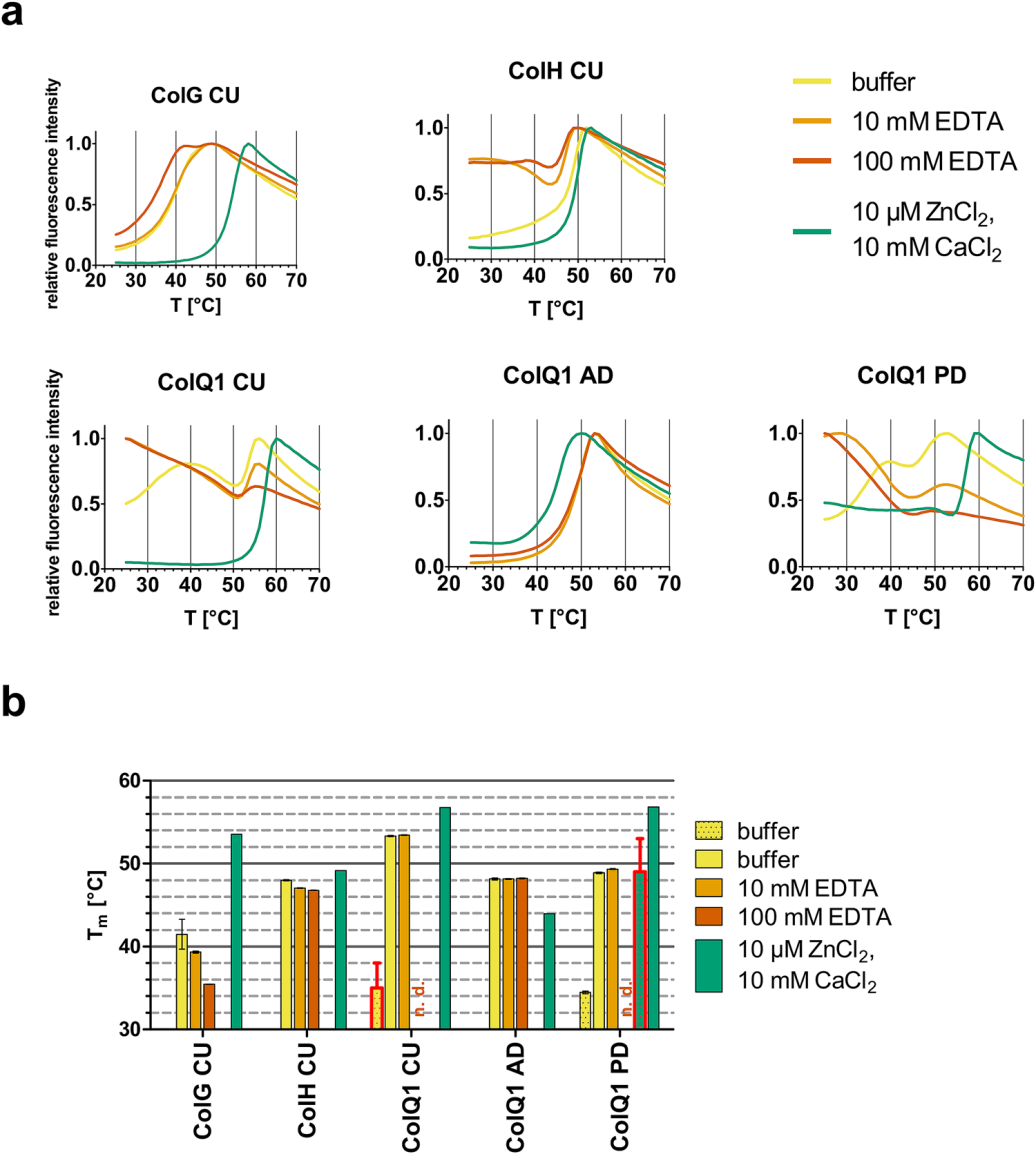
Differential scanning fluorimetry (DSF) of collagenase units of ColG, ColH and ColQ1 and activator and peptidase domains of ColQ1. (**a**) Representative melting curves in 20 mM HEPES, 80 mM NaCl, pH 7.5, 5 × SYPRO orange dye without further additives (yellow), with 10 mM EDTA added (orange), with 100 mM EDTA added (dark orange) and with 10 µM ZnCl_2_ and 10 mM CaCl_2_ added (green). (**b**) Melting temperatures determined from the DSF curves by Boltzmann fitting. n. d.: no melting temperature determination possible. In case of ColQ1 CU & PD the dotted and unfilled buffer bars are the two distinct first melting temperatures seen in (**a**). The melting temperatures that could not be determined numerically, but only graphically, are highlighted by bold red borders. Data are shown as mean ± SD (n = 3).

To verify the correct folding of recombinantly expressed ColQ1 and because *B. cereus* strain Q1 was reportedly isolated from the extreme conditions of a subsurface oil well^34^, i.e. high temperature and pressure, we compared the melting temperature (T_m_) to that of ColG and ColH (Fig. 3).

Figure 3a shows the melting curves of five different collagenase constructs: the comparison of the three collagenase units of ColG, ColH and ColQ1 as well as the activator domain and peptidase domain of ColQ1. ColG CU exhibited a monophasic unfolding in all conditions, with melting temperatures of 41.5 °C without addition or chelation of metal ions (“buffer”—yellow curve and bar), i.e. with potentially residual calcium and zinc bound from the purification and storage conditions. Upon addition of 10 and 100 mM EDTA—a chelator of divalent metals—the melting temperature was lowered to 39.3 and 35.4 °C, respectively (light and dark orange curve/bar). After addition of 10 mM CaCl_2_ and 10 µM ZnCl_2_ the T_m_ rose by more than 10 °C compared to the buffer control to 53.5 °C (green curve/bar). This indicates that that the two domains of the ColG CU, the activator and the peptidase domains, exhibit similar thermal stabilities or unfold in concert.

Similar as ColG CU, ColH CU showed a monophasic melting behaviour in the HEPES-buffered saline (“buffer”) and upon addition of Ca^2+^ and Zn^2+^, with very similar T_m_s of 48.0 °C and 49.2 °C, respectively. Upon addition of EDTA the melting behaviour became more complex, with a high starting signal followed by a melting point at 47.1 °C (10 mM EDTA) or 46.8 °C (100 mM EDTA). This indicates that under metal-chelating conditions one domain was unfolded from the beginning on and a second domain unfolded at higher temperature.

In stark contrast to ColG and ColH, ColQ1 CU gave a pronounced biphasic melting curve in the absence of additional metals or metal chelator. The first melting point could be only graphically estimated to approximately 30 °C, the inflection point (i.e. the melting temperature) of the second unfolding could be numerically fitted with a T_m_ of 53.3 °C. This indicates that there were two domains or subdomains present that unfold independently from each other. Upon addition of EDTA this uncoupled two-step unfolding became even more pronounced with very high starting signals (reflecting molten globule-like state in one sub-domain) followed by a melting point at 53.4 °C (orange line in Fig. 3a) and a melting point at approximately the same temperature for 100 mM EDTA that is too shallow to perform an accurate Boltzmann fit. Remarkably, upon addition of Ca^2+^ and Zn^2+^ ColQ1 CU showed a monophasic unfolding with a T_m_ of 56.8 °C, indicating not only a stabilizing effect but also a structural coupling of the folding domains. We hypothesised whether the biphasic unfolding of ColQ1 could be mapped to its activator domain and peptidase domain, cf. Fig. 1a. To test this hypothesis, we investigated the thermal stability of the individual AD and PD domains. ColQ1 AD did not show this metal dependent biphasic unfolding, but instead gave a monophasic melting curve in each condition with a T_m_ of 48.2 °C in the buffer saline and with addition of 10 and 100 mM EDTA. The addition of Ca^2+^ and Zn^2+^ lowered the T_m_ by approximately 4 °C to 43.9 °C. Importantly, however, ColQ1 PD exhibited a biphasic unfolding in all buffer conditions, which was particularly pronounced in the control buffer and when EDTA was present. In the buffer condition the melting temperatures were 34.5 and 48.9 °C for the observed two sequential unfolding events. With addition of 10 and 100 mM EDTA the curves showed high initial fluorescence, indicating that the first (sub-)domain was unfolded from the beginning on. With 10 mM EDTA present the second T_m_ could still be determined to be 49.3 °C. However in the presence of 100 mM EDTA, the transition became too shallow for an inflection point to be determined numerically. With both Ca^2+^ and Zn^2+^ present, the first unfolding event in the biphasic profile almost vanished. Its melting point could only be graphically estimated to approximately 49 °C, while the second dominant transition exhibited a T_m_ of 56.8 °C. The thermal unfolding of the ColQ1 peptidase domain thus qualitatively and quantitatively resembles the bimodal unfolding of the ColQ1 collagenase unit.

Under ideal conditions (i.e. with Ca^2+^ and Zn^2+^ supplied in the buffer), ColQ1 CU therefore had the highest T_m_ out of these three collagenases, but it also exhibited the strongest dependence on metals for stability.

### Enzymatic activity

**Figure 4:**
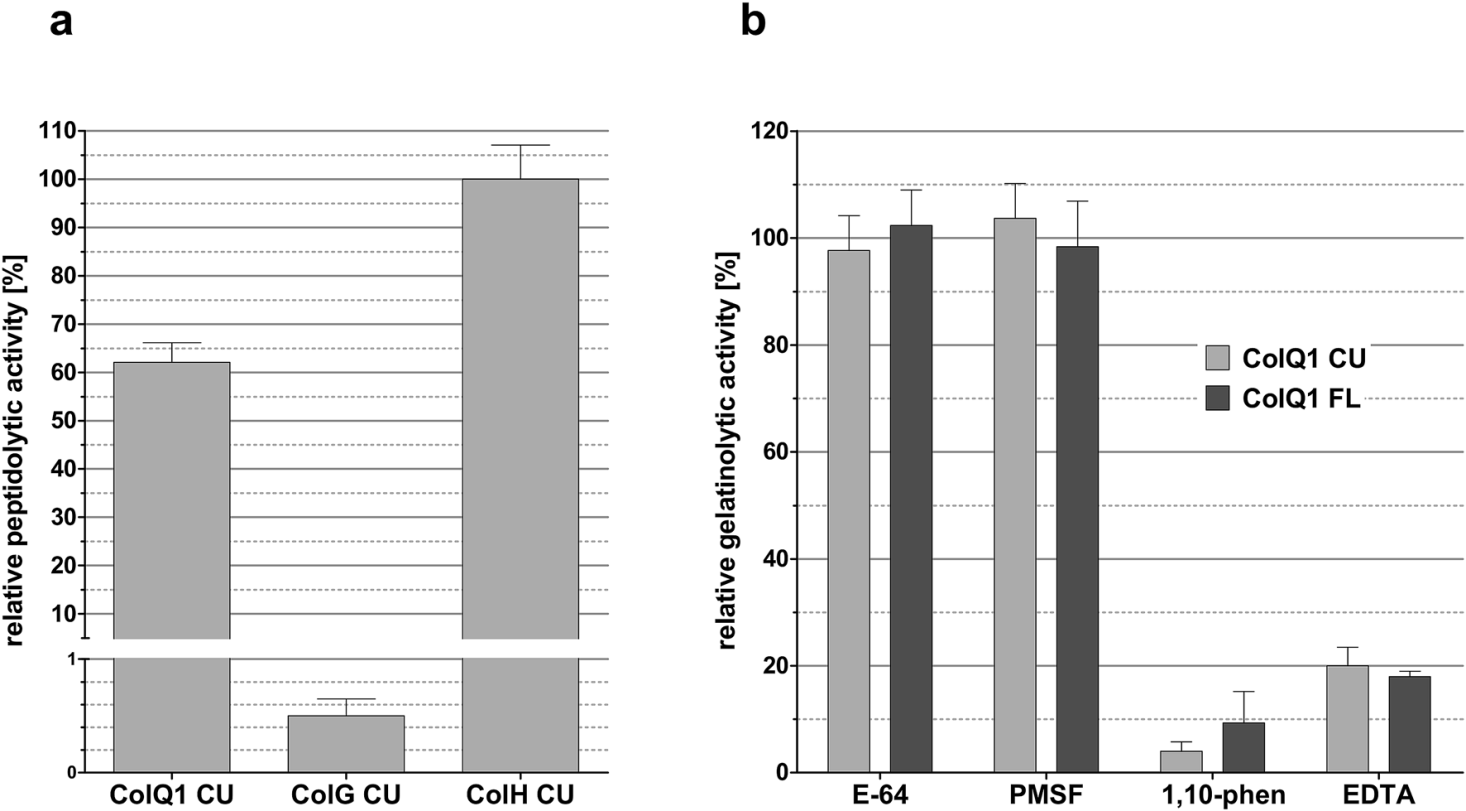
Initial enzymatic characterization of ColQ1. (**a**) Relative peptidolytic activities of ColQ1 CU, ColG CU and ColH CU against the collagenase-specific peptide substrate FALGPA. Data shows mean ± SD (ColH CU: n = 3, ColG CU/ColQ1 CU: n = 5). (**b**) Relative activities of ColQ1 FL and CU against fluorescein-labelled gelatin (DQ gelatin, Molecular Probes) after incubation with cysteine- and serine protease inhibitors E-64 and PMSF and metalloprotease inhibitors 1,10-phenanthroline and EDTA. Data are graphed as mean ± SD (n = 3).

To test whether this novel protein has collagenase-like enzymatic activity, we assayed the peptidase activity using the collagen-like peptidic substrate N-[3-(2-Furyl)acryloyl]-Leu-Gly-Pro-Ala (FALGPA) (Fig. 4a). As reported previously, among the clostridial collagenases ColH has the highest activity towards this substrate while ColG has the lowest turnover^41^. The putative bacillial collagenase ColQ1 indeed showed activity towards FALGPA with approximately 60% of the reaction velocity of ColH and over 100-fold higher velocity than ColG.

To put the computational annotation as zinc-metallopeptidase to the test, we measured the activity of ColQ1 (CU and FL) towards the relatively simple substrate gelatin in the presence of different inhibitors (Fig. 4b). We tested E-64 and PMSF, broad-spectrum cysteine and serine protease inhibitors, respectively. Both inhibitors did not inhibit ColQ1. It is therefore highly unlikely that this enzyme is either a cysteine or a serine protease. EDTA, which also was detrimental to correct folding, and the more zinc-specific chelator 1,10-phenanthroline^42^ strongly inhibited the reaction.

**Figure 5:**
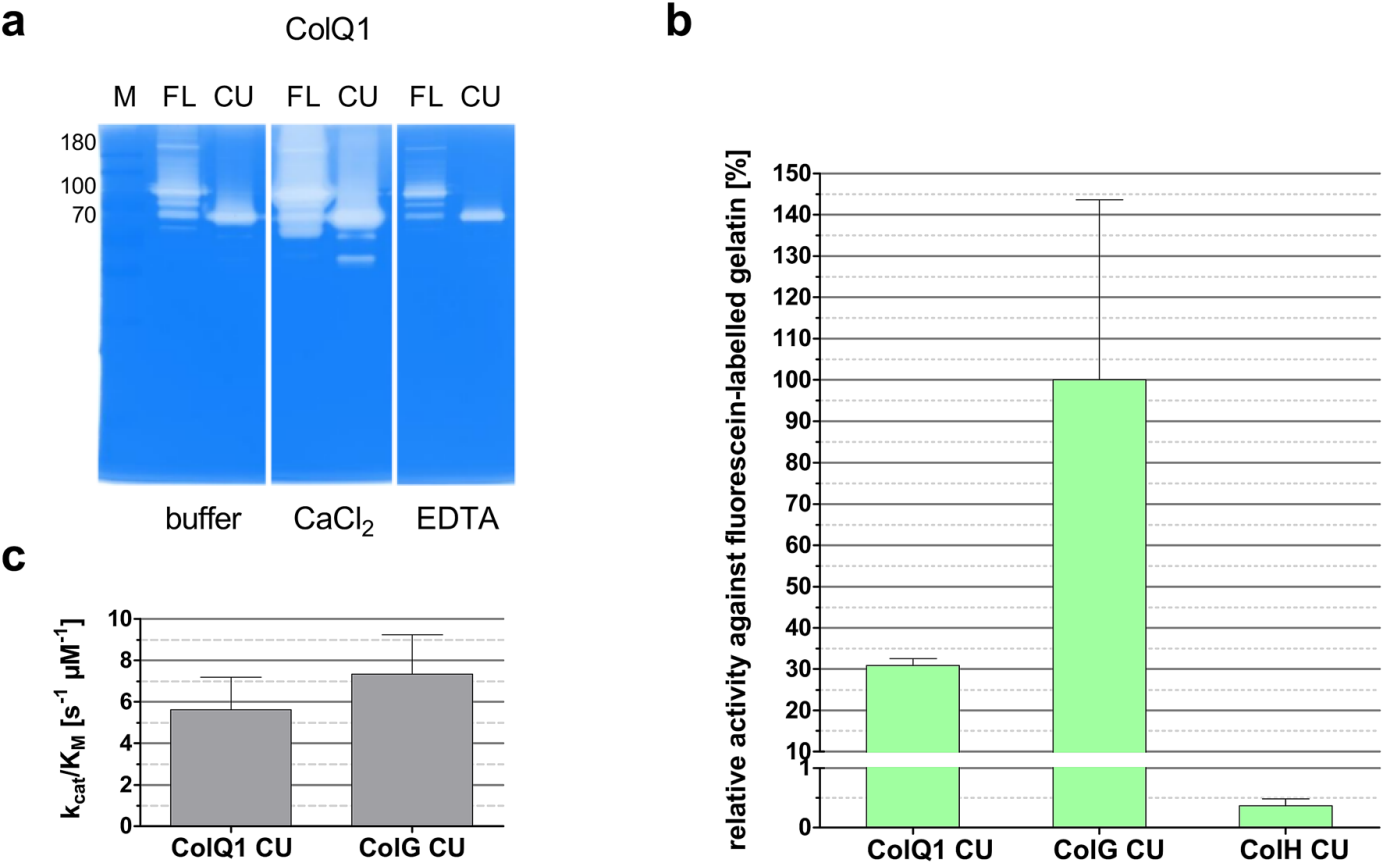
Activity of bacterial collagenases against gelatin (**a**) Zymogram showing the gelatinolytic activity of ColQ1 FL and CU in developing buffer without additives, with 5 mM EDTA and with 5 mM CaCl_2_ added. Clear regions against blue background indicate gelatin in the gel has been cleaved. (**b**) Activity of the collagenase units of ColG, H and Q1 against fluorescein-labelled gelatin (DQ gelatin, Molecular Probes). (**c**) Catalytic efficiency (k_cat_/K_M_) of ColG CU and ColQ1 CU towards gelatin from rat tail determined by discontinuous degradation assay using fluorescamine detection of neo-N-termini. Error bars indicate standard error of nonlinear Michaelis–Menten fit.

These results show that ColQ1 is indeed an active metallopeptidase with a substrate preference for collagen-typical sequences. Consequently, we wanted to characterize the activity more comprehensively, especially in comparison to the well-characterised clostridial collagenases. Figure 5 shows different experiments exploring ColQ1’s gelatinolytic activity. A representative gelatin zymogram with ColQ1 FL and CU is shown in Fig. 5a. Clear bands on the Coomassie-stained SDS-PAGE gel indicate that the enzyme cleaved the gelatin copolymerized into the gel. Consistent with its metal dependence, ColQ1 FL and CU activities were reduced by EDTA and increased by the addition of CaCl_2_ in the developing buffer. The gelatinolytic activities clearly peaked at monomeric bands, but were also visible at higher molecular weight, suggesting a partially retarded migration of ColQ1 FL through the gelatin-containing SDS gel. Figure 5b compares the activity of ColQ1 CU towards fluorescein-labelled gelatin with that of ColG CU and ColH CU at one given substrate concentration. ColQ1 CU had approximately one third of the activity of ColG CU and 60 times more activity than ColH CU. Since the gelatinolytic activity was in a similar range as that of ColG CU we compared these two enzymes more comprehensively using Michaelis–Menten kinetics. To ensure the physiological relevance of the results, we used gelatin derived from unlabelled type I collagen from rat tail as the substrate. Initial velocities were determined using a discontinuous cleavage assay where the substrate was mixed with enzyme, samples were taken at set time points and the reaction was stopped by addition of EDTA. The progress was then quantified by reaction of primary amines with fluorescamine, which can be detected by fluorescence. After subtraction of the signal for uncleaved collagen this gives an accurate quantification of neo-N-termini generated by enzymatic cleavage (Fig. 5c). ColG CU and ColQ1 CU displayed similar kinetics of native gelatin degradation. Both enzymes exhibited a virtually identical affinity towards gelatin with a K_M_ of approximately 1.4 µM (ColG CU: 1.35 ± 0.3 µM; ColQ1 CU: 1.36 ± 0.3 µM) and k_cat_ values of 9.9 ± 0.7 and 7.7 ± 0.5 s^−1^, respectively. Accordingly, the gelatin turnover (k_cat_/K_M_) was slightly lower for ColQ1 CU than for ColG CU (ColG CU: 7.3 ± 1.9 s^−1^ µM^−1^; ColQ1 CU: 5.6 ± 1.6 s^−1^ µM^−1^).

From these data (Figs. 4, 5) we conclude that ColQ1, annotated as a member of the gluzincin family M9 and a sequence homologue of the clostridial collagenases ColG, H and T, is indeed a calcium-dependent zinc-metalloprotease with specificity for collagen-derived peptide sequences.

**Figure 6:**
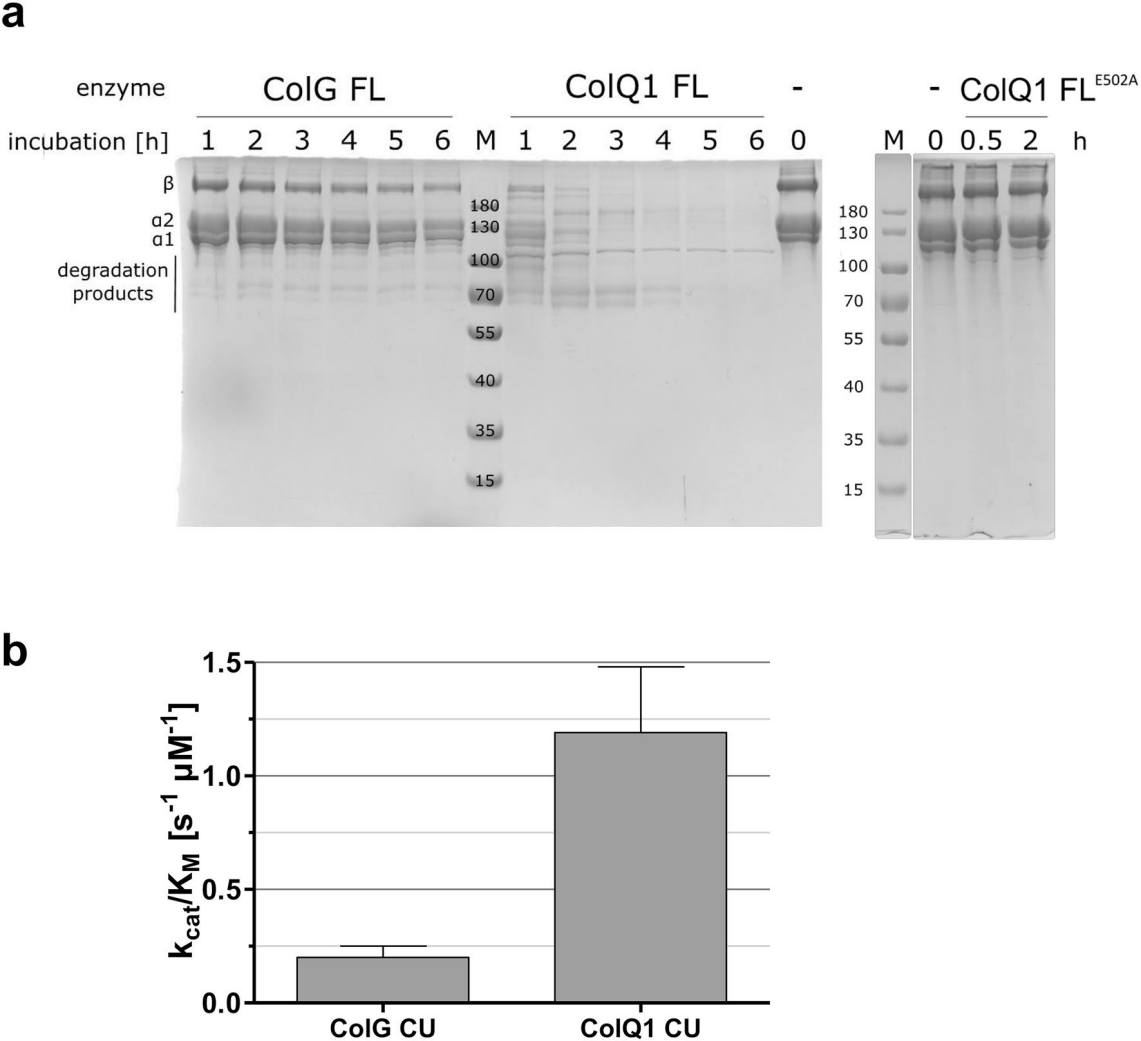
Activity of bacterial collagenases against collagen. (**a**) Collagenolytic activity of ColG and ColQ1 visualized on SDS-PAGE. M: molecular weight standards, ColG FL, ColQ1 FL and ColQ1 FL E502A (catalytic dead mutant) were incubated with 1 mg/ml type I collagen for the indicated times, 0: 1 mg/ml type I collagen without any protease. (**b**) Catalytic efficiency (k_cat_/K_M_) of ColG CU and ColQ1 CU towards type I collagen from rat tail determined by discontinuous degradation assay using fluorescamine detection of neo-N-termini. Error bars indicate standard error of nonlinear Michaelis–Menten fit.

The hallmark of a true collagenase is the ability to cleave natively folded triple-helical collagen under physiological conditions^43^. We therefore incubated ColQ1 FL and ColG FL with bovine type I collagen for up to 6 h at 25 °C. Type I (atelo-) collagen comprises exclusively triple-helical content and in its native conformation it cannot be processed by most proteases. ColG FL cleaved approximately half of the collagen in the reaction mixture within 6 h. In contrast, ColQ1 FL degraded the majority of the collagen already after one hour and after 5 h there was nearly no collagen visible in the Coomassie-stained SDS-PAGE (Fig. 6a). We can therefore conclude that ColQ1 is a true collagenase. In fact, it processes collagen much faster than ColG. As a control, we incubated collagen with a ColQ1 FL mutant in which the conserved glutamate of the HEXXH-motif characteristic for zinc-metalloproteases was changed to alanine (ColQ1 FL^E502A^). By homology, this glutamate serves as the general acid/base in catalysis^15,16^. This mutant was not able to cleave collagen (Fig. 6a), further validating the annotation as a zinc-metalloprotease.

To avoid the intricacies of quantifying collagen-derived product degradation assays monitored by SDS-PAGE, we further investigated the kinetics of collagen degradation in the same discontinuous assay as described for gelatin (Fig. 6b). We used the same type I collagen from rat tail; the substrates used in the gelatinolytic and collagenolytic assays therefore shared the same primary sequence. Collagen was used in its natively folded form while gelatin was prepared by collagen denaturation.

Using native collagen as a substrate, both enzymes exhibited substantially lower turnover rates than for gelatin, with catalytic efficiencies (k_cat_/K_M_) of 0.2 ± 0.05 (ColG CU) and 1.19 ± 0.29 (ColQ1 CU) s^−1^ µM^−1^ for collagen, as could be expected^12^. The only difference between the substrates gelatin and collagen is the triple-helical fold. Assuming a two-step processing of collagen, this comparison implies that the rate-limiting step in collagenolysis for these enzymes must be related to the unwinding of the triple helix.

With a K_M_ of 1.72 ± 0.4 µM and a k_cat_ of 2.1 ± 0.13 s^−1^, ColQ1 CU displayed a two-fold higher affinity towards collagen than ColG CU and a three-fold higher turnover rate (ColG K_M_: 3.40 ± 0.7 µM; ColG k_cat_: 0.70 ± 0.05 s^−1^). In effect, we found that ColQ1 CU can process folded collagen 6 times more efficiently than ColG CU.

## Discussion

We have shown here that the general strategy for the high-yield expression and purification of clostridial collagenases in *E. coli* presented by Ducka et al.^40^ is also applicable to homologous collagenases from bacilli with only minor modifications. This approach yields mg-amounts of highly pure and monodisperse protein suitable for biochemical and enzymatic characterization.

As a first step towards a more comprehensive characterization of non-clostridial bacterial collagenases we have shown here—by the use of specific inhibitors and through mutation of the conserved zinc-orienting glutamate residue in the HEXXH-motif—that this novel enzyme from *B. cereus* strain Q1 is indeed a metalloprotease of the gluzincin class, as the bioinformatic annotation suggests. Its activity against triple-helical type I collagen under physiological buffer conditions shows that this enzyme is a true collagenase^43^.

In fact, ColQ1 is much more active against triple-helical collagen than ColG, the clostridial collagenase with the highest reported collagenolytic activity^12^. This is consistent with our recent report on the activity of ColA from *B. cereus* type strain ATCC 14579^28^ and indicates that, although historically less studied and therefore much less well described, bacillial collagenases may in general be much more efficient collagenolytic enzymes than clostridial collagenases. By determination of the kinetics towards collagen and its thermally denatured variant gelatin we showed that this difference can be explained by a higher helicase activity of ColQ1. The molecular mechanism for this high helicase activity cannot be fully explained by the data available at present and will have to be investigated further.

The to our knowledge only other comprehensive kinetic characterization of collagenolysis by ColG (termed β-collagenase in older publications) was conducted by Mallya et al.^44^ and determined a K_M_ of 12 µM and a k_cat_ of 1000 h^−1^ (≈ 0.28 s^−1^) towards soluble type I collagen from rat tail at 25 °C. These results, while not identical, are in a similar range as the results presented in this study. Although the substrate used here is very similar (acetylated, soluble type I collagen from rat tails in a neutral buffer) there are some significant differences that can account for the slightly different results. Firstly, the enzyme was prepared in a very different manner. While we expressed the collagenases recombinantly in *E. coli*, in the previous study it was isolated from commercial *C. histolyticum* collagenase preparation by a series of chromatographic steps. Secondly, previous enzymatic reactions were carried out in 50 mM Tricine, 200 mM NaCl, 10 mM CaCl_2_, pH 7.5 and we now know that (1) both calcium and zinc are essential for correct folding and activity of clostridial collagenases and (2) tricine can act as a chelator of divalent metal ions^45^ and could therefore have influenced the activity, unlike the HEPES buffer used in the present study. Furthermore, in the previous study precipitation with 50% dioxane was used to remove uncleaved substrate. This was not necessary in our experimental design. Taking these experimental differences into account, the kinetic data determined here match the available literature reasonably well.

**Figure 7:**
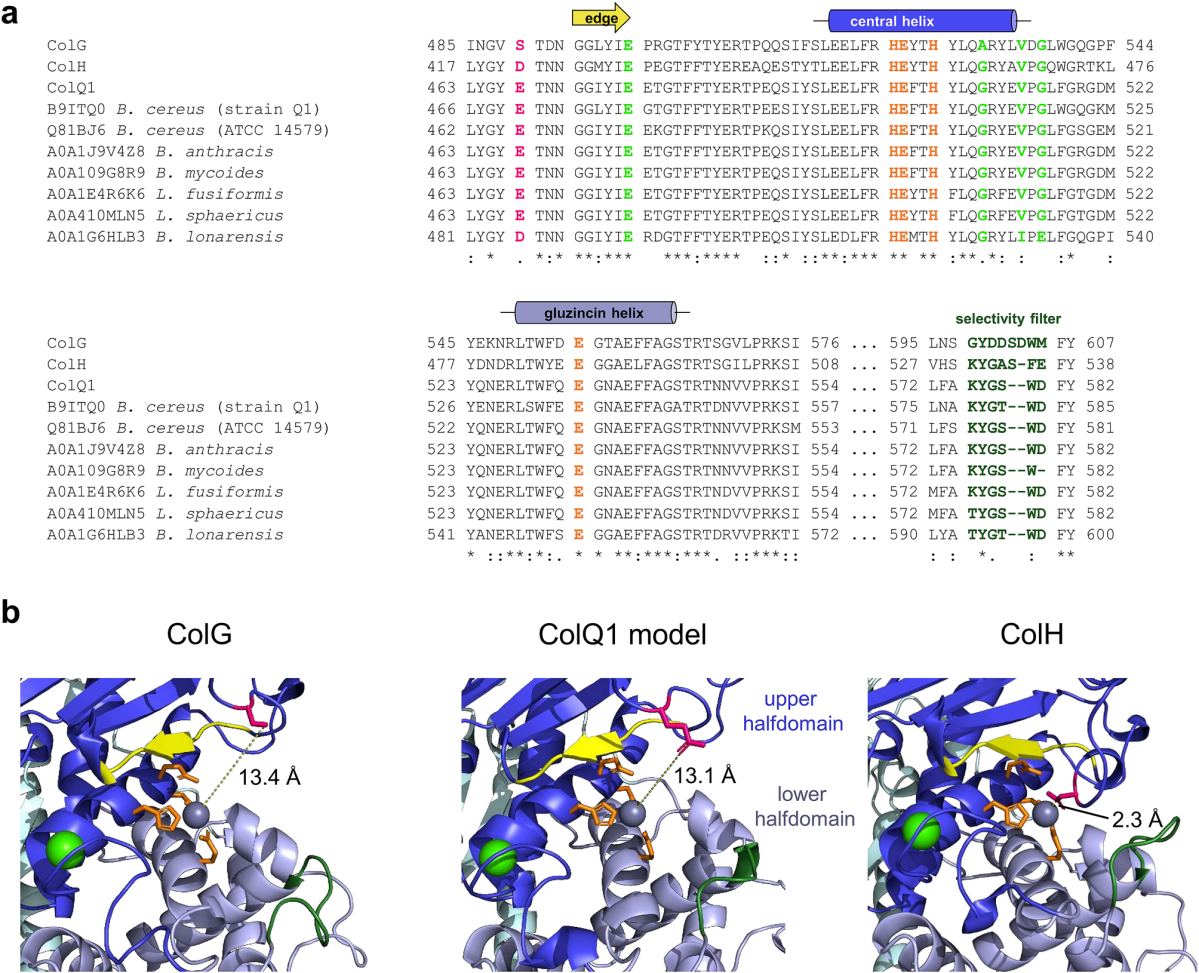
Comparison of catalytic centres of bacterial collagenases (**a**) Alignment of the catalytic centers of several clostridial and bacillial collagenases. ColG (Q9X721) and ColH (Q46085) from *C. histolyticum*, ColQ1 (B9J3S4) from *B. cereus* strain Q1, second collagenase (B9ITQ0) from *B. cereus* strain Q1, ColA (Q81BJ6) from *B. cereus* strain ATCC 14579, and one collagenase each from *B. anthracis, B. mycoides*, *B. lonarensis*, *Lysinibacillus fusiformis* and *L*. *sphaericus* (UniProt accession numbers indicated within figure). Structural annotations (edge strand in yellow, central helix in blue and gluzincin helix in light blue) are based on Eckhard et al.^15^. The alignment was aided by Clustal Omega^75^. Calcium-binding residues, zinc-binding residues and the selectivity filter are shown in light green, orange and dark green, respectively. The zinc-stabilizing aspartate (in ColH) and homologous residues in the other collagenases are highlighted in pink. (**b**) Close-up on the active sites of ColG (PDB: 4ARE), ColH (PDB: 4AR1) and the homology model of ColQ1. Modelling was performed using Modeller^58^. The upper half-domain, lower half-domain, calcium and zinc are shown in dark blue, light blue, light green and grey, respectively. Colours for the highlighted structural motifs and metal-binding residues are the same as in (**a**).

Clostridial collagenases are traditionally divided into class I collagenases with high collagenolytic activity (ColG) and class II collagenases with high peptidolytic activity (ColH)^12^. Its high collagenolytic and gelatinolytic activity would make ColQ1 a class I collagenase. However, ColQ1 also shows remarkably high peptidolytic activity, comparable to that of class II collagenase ColH. This raises the question whether this division into collagenolytic class I and peptidolytic class II collagenases is applicable to non-clostridial collagenases. A possible explanation for the high peptidolytic activity of ColQ1 can be found in a comparison of the catalytic centres of clostridial and bacillial collagenases (Fig. 7) based on the crystal structures of ColH PD and ColG CU and a homology model of ColQ1 PD. As reported previously^15^, ColH has an additional zinc-coordinating residue (aspartate 421) in the upper catalytic half-domain, shortly before the substrate-binding edge strand, in a position where the other clostridial collagenases have a serine. This helps ColH to maintain a more rigid and stable catalytic centre, even in the absence of a complex substrate like gelatin or collagen to stabilize it. By contrast, ColG’s active site has a low affinity to the catalytic zinc, accompanied by relatively high local flexibility^15^. ColQ1 and many other closely related putative collagenases have a glutamate in the position equivalent to Asp421 in ColH (Fig. 7, highlighted in pink), like aspartate a negatively charged amino acid side chain that could be expected to similarly stabilize the catalytic Zn^2+^ ion. Although this glutamate is located too far away from the catalytic zinc to interact with it in a homology model (Fig. 7b), it is conceivable that the loop region that contains it is flexible enough to change its position depending on the substrate that is bound. This loop could then “flip up” in order to enable collagen binding or “flip down” into a position resembling the catalytic centre of ColH to facilitate efficient peptidolytic activity. A conformational change like this could in part explain how *Bacillus* collagenases manage to straddle this dual high activity against peptidic and more complex substrates. Furthermore, the amino acid sequence of the selectivity filter—a loop region located approximately 50 amino acids downstream of the gluzincin-helix, forming a sort of lower lip in the catalytic centre^15^—resembles that of ColH more closely than that of ColG, yet in the structural homology model it more closely resembles the configuration found in ColG. It is however not identical to that of ColH but seems to be strongly conserved among the *Bacillus* and *Lysinibacillus* (putative) collagenases.

Given the dynamic nature of collagen substrate recognition and turnover by collagenases, it is interesting to analyse the thermally induced, metal-dependent unfolding of collagenases. The clostridial collagenases ColG and ColH both exhibited a monophasic unfolding behaviour, but differed strikingly in their metal dependence, cf. Fig. 3. The pronounced stabilization of ColG by the presence of zinc and calcium reflects its low metal affinity, whereas ColH was shown to have its catalytic zinc (and calcium) site always occupied^16^. The low zinc occupancy in ColG readily explains why it exhibits a low peptidolytic activity (Fig. 4).

**Figure 8:**
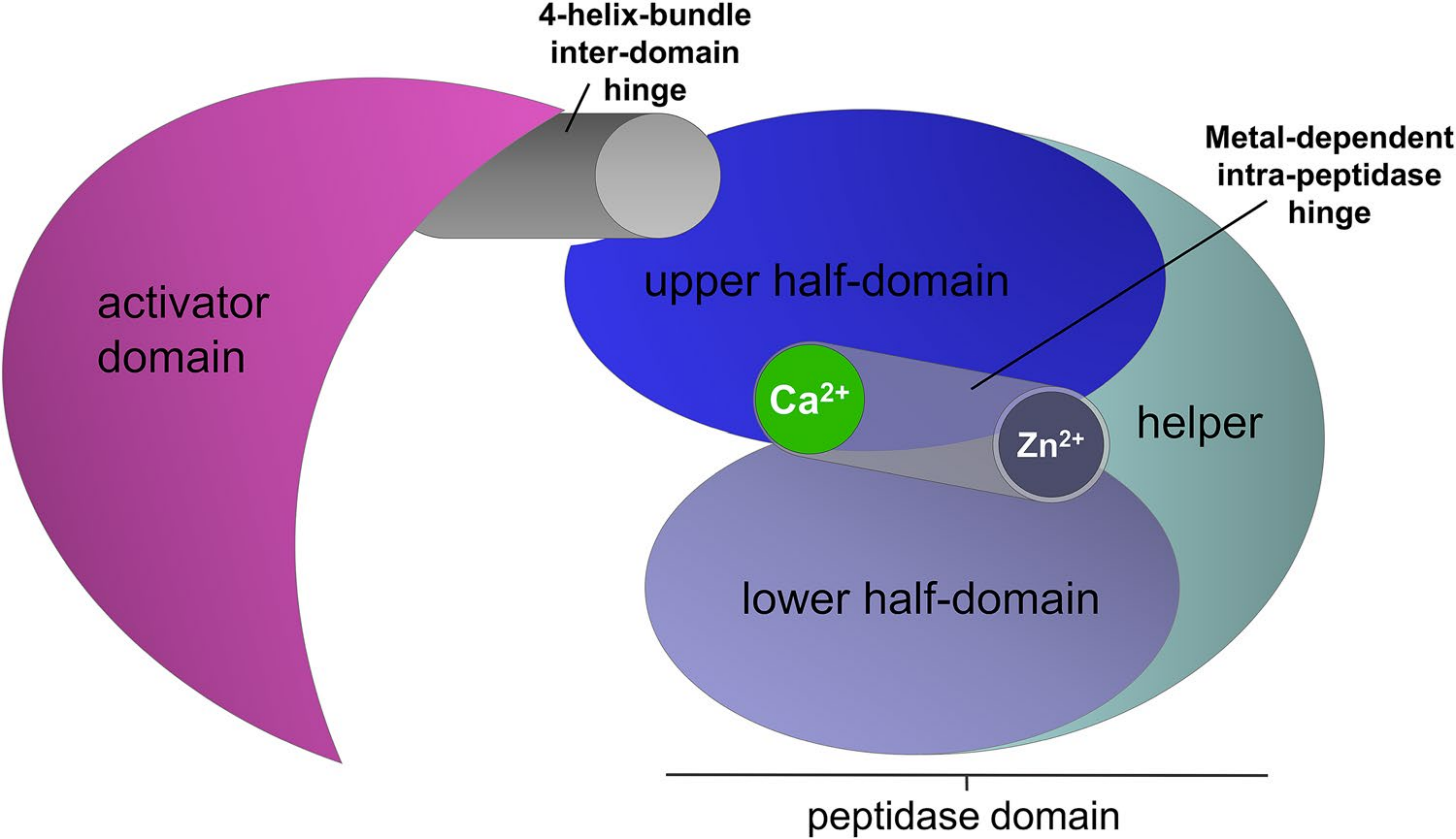
Schematic illustration of the hinge elements for collagenolytic, gelatinolytic and peptidolytic activities in bacterial collagenases.

ColQ1 shares with ColG a strong metal dependence in its thermal stability. However, contrasting both ColG and ColH, the bacillial ColQ1 exhibited a pronounced biphasic unfolding, a behaviour characteristic for a two-domain protein. We questioned whether the two discrete unfolding events could be related to the activator domain and peptidase domain. Surprisingly, however, we found the isolated peptidase domain to account for the biphasic unfolding seen in the collagenase unit of ColQ1. The peptidase domain itself is segmented into an upper and lower half-domain. At standard buffer conditions these two half-domains unfold separately, however upon addition of 10 µM ZnCl_2_ and 10 mM CaCl_2_ the unfolding of both domains is (1) stabilized and (2) coupled. Only a minor fraction unfolds prematurely, while the majority denatures cooperatively at 56.8 °C. The coupling of the (un-)folding of the lower and upper half-domains of the peptidase by Ca^2+^ and Zn^2+^ is consistent with their binding sites being placed at the interface of both half-domains, cf. Figure 7b. This metal-dependent hinge motion within the peptidase domain complements the functionally critical hinge between the activator domain and peptidase domain. The latter is formed by a four-helix bundle and is required for collagen degradation^16^. One way to interpret the strong collagenase activity in ColG and in particular in ColQ1 is that both the four-helix hinge and the metal hinge (Fig. 8) are required for efficient collagen recognition and processing. By contrast, the metal hinge appears much stiffer in ColH, which may relate to its significantly reduced collagenolytic activity. It is also possible that the helper subdomain plays an as yet unclear role in regulating the opposing activities towards complex and simple peptide substrates.

Although ColQ1 originates from a *B. cereus* strain isolated from a deep-surface oil reservoir^34^, in which temperatures can rise on average by 3 °C per 100 m in depth^46^ we found no pronounced thermophilicity in ColQ1 on the level of the collagenase unit. The highest melting temperature observed was 56.8 °C for ColQ1 CU compared to 49.2 °C and 53.5 °C for ColH CU and ColG CU in the presence of ions, respectively. This temperature fails to meet the thermal stability range of 80–100 °C for thermophilic proteins^47^. Yet the mesophilic nature of ColQ1 CU complies with its physiological function. There is no obvious rationale for the evolution of a thermophilic collagenase, as its physiological substrate collagen starts to unfold at 37°C^48^ and can then be degraded by various proteases (e.g. MM-1, -2, -3, -9, -10^48^, cathepsin B and K^49^, pepsin, trypsin^50^).

While there are no reports that implicate bacillial collagenases as virulence factors in *B. cereus*-associated foodborne diseases, in case of *B. cereus* mediated endophthalmitis^32^ and periodontal diseases, however, collagenases have been found in the isolates of vitreous humor and dental plaques^32,33,51,52^. Considering the high collagenolytic efficiency of bacillial collagenases, as shown here for ColQ1 and for *B. cereus* ATCC 14579 ColA^28^, we propose that these enzymes facilitate the infiltration of the target tissues by degradation of the protective surrounding extracellular matrix, which then allows the diffusion of other virulence factors into the tissue. Beecher et al.^32^, for example, observed a progressive degradation of the collagenous lens capsule prior to colonization of the lens cortex by *B. cereus*. The authors found a bacillial collagenase in the vitreous fluid of infected eyes, suggesting that it had breached the collagen barrier and thus enabled the entry of the bacteria. Yet, further research into the role of bacillial collagenases as virulence factor in this pathogenic settings is required.

In conclusion, we have shown here the recombinant expression and a comprehensive biophysical and enzymatic characterization of a novel collagenolytic enzyme from *B. cereus* strain Q1. We demonstrated that this collagenase (and potentially bacillial collagenases in general) have a remarkably high collagenolytic activity compared to the well-described clostridial collagenases. Since clostridial collagenases are already used in medical and biotechnological applications, such as the treatment of Dupuytren’s contracture and in the food and leather industries^21,22,26,53^, this highly active homologue could present an attractive improvement in these areas. Furthermore, we have shown for the first time that the rate-limiting step in bacterial collagenolysis is the unwinding of the triple helix, i.e. the helicase activity.

## Methods

### Bioinformatic analysis

Signal peptide prediction was carried out using the SignalP 4.1 webserver^39^ and domain predictions were carried out using InterPro and Pfam^54,55^. Protein sequence alignments were performed with Blastp^56^ using the protein sequence of ColA from *B. cereus* strain Q1 (Uniprot accession B9J3S4) as the query and ColG (Q9X721), ColH (Q46085), ColT (Q899Y1) and ColA from *B. cereus* strain ATCC 14579 (Q81BJ6) as subject sequences. For alignment of the separate domains, domain boundaries of the three clostridial collagenases were taken from Eckhard et al., 2013^15^, while domain boundaries for ColQ1 and ColA were inferred from the abovementioned domain predictions and sequence homology to the clostridial collagenases (see Supplementary Table S1). Modelling of the ColQ1 structure was carried out using HHpred and Modeller^57,58^.

### Cloning

The coding DNA sequences were amplified by polymerase chain reaction using synthesized plasmid DNA (*E. coli* codon-optimized full-length sequence of ColQ1 Eurofins Genomics, Ebersberg, Germany) as template and the appropriate primers (see Supplementary Table S2, Eurofins Genomics) containing the restriction enzyme sites BamHI and KpnI for cloning the insert into a modified pET15b vector containing an N-terminal His_6_-tag. The PCR products were purified using the GeneJET PCR Purification kit (Thermo Fisher Scientific, Waltham, USA). Vector and PCR products were digested with the appropriate restriction enzymes (FD BamHI/KpnI, Thermo Fisher Scientific, Waltham, USA), purified via preparative DNA electrophoresis followed by gel extraction using the MinElute Gel Extraction Kit (Qiagen, Hilden, Germany), ligated and electrocompetent XL2blue cells (Stratagene, San Diego, USA) were transformed with the resulting plasmids following standard protocols. The E502A mutation was introduced via inverse PCR^59^. All constructs were verified by sequencing at Eurofins Genomics.

### Recombinant protein expression and purification

ColG variants and ColH collagenase unit were expressed and purified as described previously^40^. Chemocompetent NiCo21(DE3) (New England Biolabs, Ipswich, USA) cells were transformed with *E. coli* codon-optimized plasmids encoding for the full-length mature and the collagenase unit of ColQ1 wild-type and catalytically dead mutants. Starter cultures were inoculated with a single clone from a fresh LB plate containing 100 µg/ml ampicillin and grown over night at 37 °C and 230 rpm. Expression cultures were inoculated 1:500 with starter culture and grown at 37 °C and 230 rpm in baffled flasks. At OD_600_ of 0.3–0.4 NaCl at a resulting concentration of 0.25 M was added to promote accumulation of osmolytes and thereby enhance solubility of the recombinant protein^60^. Protein expression was induced in the early stationary phase (OD_600_ 1.0–1.2) by addition of 0.5 mM IPTG; at the same time 1 mM CaCl_2_ was added. Protein was expressed over night at 17 or 25 °C for the full-length enzyme and collagenase unit, respectively. The cells were collected by centrifugation at 4,000 g and 4 °C for 15 min and the periplasm was released by osmotic shock treatment^61^. Cells were resuspended in 50 mM NaH_2_PO_4_, 300 mM NaCl, 10 mM imidazole, 10 mM MgCl_2_ pH 8.0 containing DNAseI (AppliChem, Darmstadt, Germany) and disrupted by sonication on ice (5 × 45 s, ≈45 W) using a Sonopuls HD 2070 ultrasound homogenizer (BANDELIN, Berlin, Germany). Insoluble components were removed by centrifugation at 16,000 g, 4 °C for 2 × 15 min.

IMAC was carried out in batch columns containing pre-equilibrated Ni Sepharose 6 Fast Flow resin (GE Healthcare Life Sciences, Chicago, USA) using cooled buffers. Cleared supernatants were incubated on the column for 30 min at 4 °C, the column was washed three times with 6 column volumes (CV) 50 mM NaH_2_PO_4_, 300 mM NaCl, pH 8.0 containing 25 mM imidazole and the target protein was eluted with 9 CV of the buffer above containing 250 mM imidazole and 10 mM β-mercaptoethanol. The elution fractions were concentrated and rebuffered into 50 mM NaH_2_PO_4_, 100 mM NaCl, 10 mM β-mercaptoethanol, < 10 mM imidazole, pH 7.5 by ultrafiltration. N-terminal His_6_-tags were removed by tobacco etch virus (TEV) protease (produced in-house according to van den Berg et al.^62^) cleavage at 4 °C over night in a molar ratio of 1:5 (TEV-protease to target protein). To remove TEV-protease (N-terminally His_6_-tagged) and uncleaved target protein, rechromatography on Ni Sepharose 6 Fast Flow was performed.

Size-exclusion chromatography was employed as a final polishing step at 22 °C using cooled buffer (25 mM HEPES, 100 mM NaCl, 5% glycerol, 1 mM DTT, pH 7.5). Concentrated protein was separated on a Superdex S200 10/300 GL column (GE Healthcare Life Sciences, Chicago, USA) using the ÄKTA FPLC system and fractions found to be sufficiently pure by SDS-PAGE (≥ 90%) were flash frozen in liquid nitrogen and stored at − 80 °C.

### Preparation of acetylated type I collagen from rat tails

All purification and chemical modification steps were carried out on ice or at 4 °C. Type I collagen from rat tails was isolated as described by Steffensen et al.^63^. Collagen was lyophilized and stored at − 20 °C. Acetylation of collagen was adapted from Mookhtiar et al.^64^. In short, collagen was dialyzed against 10 mM sodium borate, 0.2 M CaCl_2_, pH 9.0, acetic anhydride was added to 1% (v/v) over 1 h under gentle stirring, followed by another 30 min of stirring and dialysis into 0.1 M acetic acid. Acetylated collagen was concentrated as high as possible by ultracentrifugation and dialyzed into enzyme reaction buffer. Collagen concentrations during this procedure were routinely monitored by a modified Lowry assay^65^ and the native fold of the substrate was verified by digestion with α-chymotrypsin^66^.

### Thermal shift assay

Melting temperatures were determined in 20 mM HEPES, 80 mM NaCl, pH 7.5, 5 × SYPRO Orange dye (Molecular Probes, Eugene, USA). As indicated, 10 mM CaCl_2_ and 10 µM ZnCl_2_, 10 mM or 100 mM EDTA were added. The temperature was raised in 1 °C increments from 25 to 95 °C and fluorescence was recorded using the Applied Biosystems 7500 Real-Time PCR System (Thermo Fisher Scientific, Waltham, USA) at the SYBR Green setting. Data was analysed as described by Niesen et al.^67^ and Boltzmann fitting was performed with GraphPad Prism 5.0 (GraphPad Software, La Jolla, USA).

### Enzymatic assays

#### FALGPA

Peptidolytic activity was determined using FALGPA^68^. The assay was carried out in 100 mM HEPES, 200 mM NaCl, 10 mM CaCl_2_, 10 µM ZnCl_2_, pH 7.5 at a final FALGPA (AppliChem PanReac, Darmstadt, Germany) concentration of 3.1 mM at 25 °C. The substrate was dissolved in reaction buffer for 30 min and the concentration was verified by absorbance measurement at 305 nm (ε = 24.7 mM^−1^ cm^−1^). Final enzyme concentrations were 25 and 50 nM for ColH, 50 and 100 nM for ColQ1 and between 0.5 and 5.1 µM for ColG. Reaction progress was monitored via absorption measurement at 345 nm over 45 min in 30 s intervals in a Tecan Infinite M200 plate reader (Tecan, Grödig, Austria) and initial velocities were determined as described by Briers et al.^69^. All reactions were carried out at least in triplicate.

#### Gelatin zymography

The assay was carried out as described by Wilson et al.^70^ using 15 ng collagenase per gel lane. Zymograms were developed in 50 mM Tris-HCl, 200 mM NaCl, 0.02% Brij35, ± 5 mM CaCl_2_, ± 5 mM EDTA, pH 7.5.

#### Fluorescein-labelled gelatin

DQ gelatin from pig skin from the EnzCheck Gelatinase/Collagenase Assay kit (Molecular Probes, Eugene, USA) was used as a substrate according to the manufacturer’s instructions. The buffer was changed to 250 mM HEPES, 150 mM NaCl, 5 mM CaCl_2_, 5 µM ZnCl_2_, pH 7.5. E-64, PMSF, EDTA and 1,10-phenanthroline were added to final concentrations of 10 µM, 1 mM, 10 mM and 10 mM, respectively. The reaction was carried out in 50 µl total volume, 0.2 µg/ml final substrate concentration, 0.5–3 nM final enzyme concentration and monitored in a Tecan Infinite M200 plate reader (Tecan, Grödig, Austria) for 8 min (5 s intervals). Initial velocities were determined as described by Briers et al.^69^. All reactions were carried out at least in triplicate.

#### Collagenolytic assay monitored via SDS-PAGE

Acid-soluble type I atelocollagen from bovine hides (Cell Guidance Systems, Cambridge, UK) at a final concentration of 1 mg/ml was digested at 25 °C by 55 nM collagenase in 250 mM HEPES, 150 mM NaCl, 5 mM CaCl_2_, 5 µM ZnCl_2_, pH 7.5. Samples were taken at indicated time points, the reaction was stopped by addition of 50 mM EDTA and the degradation was visualised on 9% SDS-PAGE gels. The correct fold of the collagen was verified by incubation with 0.83 µM α-chymotrypsin (FLUKA, Buchs, Switzerland) over night.

#### Collagenolytic/gelatinolytic activity measurements monitored by fluorescamine labelling

Acetylated rat tail collagen (final concentration 3.83–0.02 mg/ml) was mixed with collagenase G and Q1 CU in concentrations ranging from 0.375 to 9 µM and from 0.06 to 1.5 µM, respectively. The reaction was carried out in 100 mM HEPES, 200 mM NaCl, 10 mM CaCl_2_, 10 µM ZnCl_2_, pH 7.5 at 25 °C. Samples were taken after 15, 45, 75, 105, 135 and 165 s, immediately stopped with 83 mM EDTA and put on ice. If necessary, samples were diluted to < 0.5 mg/ml collagen in reaction buffer containing 83 mM EDTA and detection of primary amines (i.e.—after subtraction of background—new N-termini generated by proteolytic cleavage) was carried out by reaction with fluorescamine^71^. All collagen concentrations were verified by picrosirius red assay^72^.

For gelatinolytic activity measurement acetylated rat tail collagen was denatured at 95 °C for 10 min, left to cool to 25 °C and then diluted to the desired concentrations (0.025–3 mg/ml). To avoid (partial) refolding, gelatin stocks were either used immediately after denaturation or stored at 4 °C and denatured again, as described above, directly before use. Enzyme concentrations were between 15 and 50 nM for ColG and between 10 and 100 nM for ColQ1.

Enzyme concentrations were chosen to provide reaction velocities with < 10% substrate turnover in the measured time frame at a suitable signal-to-noise ratio. All reactions were carried out at least in triplicate. Reaction velocity was plotted against substrate concentration and K_M_ and V_max_ were determined by non-linear fitting with GraphPad Prism 5.0 (GraphPad Software, La Jolla, USA).

## Supplementary Information

Supplementary Tables
