# Supplementary material for: Biochemical characterisation of a collagenase from Bacillus cereus strain Q1
Source: Sci Rep. 2021 Feb 18;11:4187. doi: 10.1038/s41598-021-83744-6 (PMC7893005; doi:10.1038/s41598-021-83744-6)
Supplement: Supplementary file 1 — Supplementary Tables [file 41598_2021_83744_MOESM1_ESM.pdf]

Supplementary Information

Biochemical characterisation of a collagenase from *Bacillus cereus* strain Q1

Authors

Isabel J. Hoppe[1], Hans Brandstetter[1]*, Esther Schönauer[1]*

[1]Department of Biosciences, University of Salzburg, 5020 Salzburg, Austria

*corresponding author

isabeljosephin.hoppe@sbg.ac.at

Esther.Schoenauer@sbg.ac.at

Johann.Brandstetter@sbg.ac.at

Supplementary Table S 1: Domain boundaries of bacterial collagenases.

	AD	PD	PKD1	PKD2	CBD1	CBD2
ColQ1	Y94-N366	D376-G765	N767-V850	-	G853-K965	-
ColA	Y93-G365	D375-	K766-V849	-	D852-K960	-
ColG	Y119-D388	D398-D790	N795-N880	-	T885-I999	N1004-K1118
ColH	V41-D320	D330-G721	N725-D810	N814-D900	Y903-R1021	-
ColT	Y57-N330	D340-K731			I755-D870	I878-N991

Supplementary Table S 2: Cloning primers used for ColQ1 constructs. Restriction enzyme sites are underlined.

Primer name	Sequence (5’→3’)
Y94_fw	ACGT<u>GGTACC</u>TATAGCATGGCCGATCTGAAC
G765_rev	CAGCC<u>GGATCC</u>TTAGCCATCATCTTTGGCGATACCATGAAAC
K965_rev	ACGT<u>GGATCC</u>TTATTTAACGCTCAGTTTATACGTGC
N366_rev	ACGT<u>GGATCC</u>TTAGTTATAGTTGGTGGTAATCTGCTCAACTGC
D376_fw	ACGT<u>GGTACC</u>ATGGACCTGGAAAAAATTCGTAAAGAAGGCAAAG
E502A_fw	GCGTTTACCCATTATCTGCAGGGTCGTTATGAAG
E502A_rev	ATGCCGAAACAGTTCTTCCAGGCTATAAATGCTC
